# An Ideal Society? Neighbors of Diverse Origins Interact to Create and Maintain Complex Mini-Organs in the Skin

**DOI:** 10.1371/journal.pbio.0030372

**Published:** 2005-11-15

**Authors:** Sarah E Millar

## Abstract

Hair follicles are the focus of this primer on mammalian skin development.

The largest organ in the body, the skin provides protection against environmental insults and dehydration, and is an important gateway for sensory input. In addition to forming an environmental barrier, the skin has evolved to produce an amazing variety of appendages, including scales, feathers, hair follicles, sweat glands, and mammary glands (Figure 1). These organs arise from embryonic skin progenitor cells and endow wide-ranging properties, including regulation of body temperature, and the ability to fly, nurse young, and attract mates. As the body's primary frontier, the skin is both vulnerable to disease, such as melanoma, and a source of therapeutic promise, harboring accessible stem cells (SCs) that can regenerate skin and potentially other organs.

**Figure 1:**
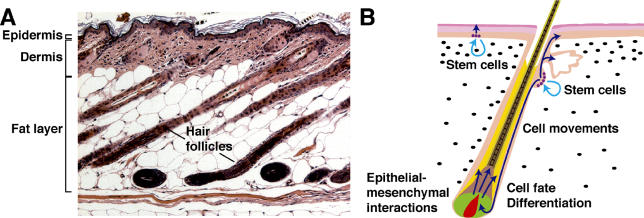
Histological and Schematic Depictions of Mature Skin (A) Hematoxylin/eosin-stained section of mouse skin at postnatal day 28, showing hair follicles in the anagen growth phase; major layers of the skin are indicated. (B) Schematic depiction of postnatal skin showing a hair follicle in the growth phase. Biological processes occurring in the skin are listed and SC locations are indicated. Dark blue arrows indicate the movements of stem and matrix cell progeny; pale blue arrows indicate SC self-renewal. Pink, epidermis and hair follicle outer root sheath; yellow, inner root sheath; green, matrix; red, hair follicle DP; light brown, hair shaft precursors; darker brown, hair shaft; violet circles, SCs; black ovals, dermal fibroblasts.

Mammalian skin contains three major cell types: epithelial cells that form a stratified epidermis containing specialized intermediate filament proteins called keratins; mesenchymal cells that form the underlying dermis and, together with epithelial cells, contribute to hair follicles and other appendages; and melanocytes that provide the skin and hair follicles with pigmentation. In addition, the skin is highly innervated, and contains populations of specialized cells including dentritic Langerhans cells (a type of antigen presenting cell), Merkel mechanoreceptor cells (which form complexes with sensory axons), and mast cells (which produce histamine). These different cell types have diverse origins, and undergo extensive interactions, migration, proliferation, and differentiation during embryonic development.

Morphogenesis is controlled by a relatively small number of key intercellular signaling pathways that occur sequentially and in specific combinatorial fashions. Activation of these pathways is directed by secreted ligands, including members of the WNT, fibroblast growth factor, tumor necrosis factor, and Hedgehog families, and bone morphogenetic protein (BMP) and other transforming growth factor superfamily members, which act over relatively short distances and bind to specific receptor proteins on neighboring cells to regulate cell shape, size, adhesion, polarity, movements, proliferation, and transcriptional activity. The molecular events underlying skin development in mammals have been studied using a variety of systems and techniques. These include analysis of human genetic syndromes and spontaneously occurring and induced mouse mutants, production of transgenic and gene-targeted mouse models, in vitro culture of rodent and human skin cells, and grafting of human or rodent skin to immune-deficient mice.

## Epidermal Origins

The epidermis originates from the outer layer of the embryo, the surface ectoderm. BMPs activate the epidermal differentiation program and induce the expression of keratin proteins via several known transcription factors [1,2]. The surface ectoderm proliferates and migrates from the dorsal midline to cover the embryo [3], and persists as a simple epithelium until approximately embryonic day (E) 9.5 of mouse embryogenesis. At this stage basal cells begin to express keratins 5 and 14, presaging epidermal stratification [2], which requires the activity of a key epidermal transcription factor that also regulates epidermal fate, proliferation, and adhesion [4–9]. By birth, the epidermis consists of a proliferative basal layer that differentiates to form suprabasal layers, and an outer, “cornified,” enucleated shell that constitutes the epidermal barrier and is continuously shed and replenished. Epidermal renewal continues throughout life, suggesting that this tissue harbors an SC population; maintenance of epidermal SCs in vivo has been recently shown to require the Rho guanosine triphosphatase Rac1 [10].

## Dermal Origins

The dermis of the skin consists of loosely packed fibroblasts and has a remarkable variety of embryonic origins. Fate mapping of mammalian dermis, which traces the developmental path of dermal cells, has been achieved by introducing artificial genes into mice. Specifically, the bacterial Cre recombinase enzyme is introduced under the control of a promoter that is active in a particular embryonic region, in this case either lateral plate mesoderm or neural crest. This transgene is combined with a Cre-activatable lacZ reporter gene, resulting in lacZ expression in Cre-expressing embryonic cells and all of their descendants. This allows tracking of the fates of these descendants by X-gal staining to detect activity of the β-galactosidase enzyme encoded by lacZ. These experiments showed that the lateral plate mesoderm gives rise to ventral trunk dermis, while head dermis arises, at least in part, from neural crest, a transient population of migratory cells derived from the neural plate [11–14]. While fate mapping of dorsal trunk dermis in mammalian embryos has not yet been described, analysis of chick–quail chimeras revealed that in avian embryos the dorsal dermis arises from the dorsal regions of somites [15,16]. Embryonic dermal fibroblasts from different body regions have different inductive properties [17–19] and show position-specific differences in gene expression, even in the adult [20]. The molecular controls of mammalian dermal development remain relatively unexplored.

**Figure 2:**
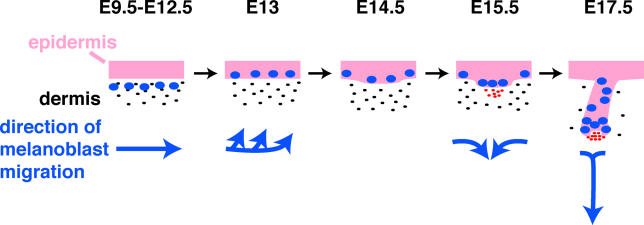
Schematic Depiction of Directions of Melanoblast Migration in Embryonic Mouse Skin from E9.5 to E17.5 Pink, epithelium; black dots, dermal fibroblasts; blue ovals, melanoblasts; red dots, dermal condensate/DP.

Pigmentation of the epidermis and hair follicles is supplied by melanocytes that manufacture melanin (black pigment) or phaeomelanin (yellow/orange pigment) and deposit it into melanosomes that are transferred to hair shaft or epidermal cells. Melanocytes differentiate from melanoblasts that arise from the neural crest. After undergoing an initial period of proliferation, melanoblasts migrate between the dermatome and overlying ectoderm, and from E10.5 of mouse embryogenesis migrate through the developing dermis. Starting at approximately E12.5, melanoblasts move from the dermis to the epidermis, and by E15.5 they begin to migrate into the developing hair follicles [21–24] (Figure 2). By two weeks of age melanoblasts are absent from haired regions of mouse epidermis [24]. In contrast, in human skin melanoblasts and melanocytes populate the epidermis as well as the hair follicles. Genetic analysis in mice has revealed requirements for several factors in melanoblast migration, proliferation, and/or survival [25,26]. Mutations in a variety of these genes are known to cause pigmentation and other neural-crest-related defects in humans as well as in mice [25].

## Interactions between Neighboring Cells Control Hair Follicle Development

**Figure 3:**
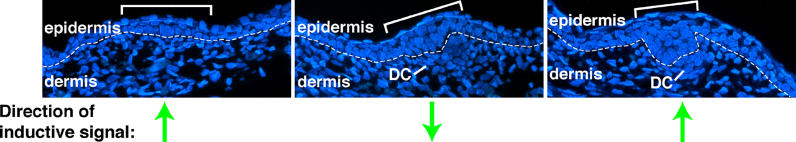
Successive Stages of Hair Follicle Development in Embryonic Mouse Skin at E14.5–E15.5 Sections were stained with Hoechst dye to reveal the nuclei. The epithelial–mesenchymal boundary is marked by a dashed white line in each panel, and sites of hair follicle development are bracketed. The directions of inductive signals are indicated for each stage (green arrows). Left: placode stage; note larger size and columnar appearance of placode nuclei compared with those in adjacent epithelium. Middle: induction of the dermal condensate (DC). Right: formation of a germ stage hair follicle with a well-developed dermal condensate.

While epidermal cells, dermal fibroblasts, and melanocytes have differing embryonic origins and migration pathways [27–29], these cells interact extensively during development of the skin and its appendages [17,19,30,31]. Experiments in which epithelium and mesenchyme from different body regions, different developmental stages, or different species were combined and allowed to develop at ectopic sites revealed that signals from the dermis initiate hair follicle development by inducing formation of a regular array of local thickenings, or placodes, in the overlying surface epithelium [17]. Signals from each epithelial placode induce the clustering of a ball of underlying mesenchymal cells, the dermal condensate. Signaling from the dermal condensate to the epithelium results in downward growth of the epithelial cells (Figure 3), which surround the dermal condensate, thereafter known as the dermal papilla (DP) [17]. The DP directs the rapid proliferation of adjacent epithelial cells called matrix cells, which gradually exit from the proliferative compartment, and terminally differentiate to form the hair shaft and the inner root sheath that molds the shaft [32]. An outer root sheath that is contiguous with the epidermis surrounds the inner root sheath, and the follicle is bound by a dermal sheath.

Surgical removal of the DP and the contiguous lower dermal sheath prevents hair growth, indicating the importance of the DP as a key signaling center for the follicle [33]. DPs or dermal sheaths implanted at ectopic sites demonstrate a remarkable ability to induce the formation of new follicles, even from terminally differentiated epithelium such as the central cornea of the eye [34–36]. This inductive ability is lost if the DP cells are cultured in the absence of epithelial cells or without the addition of WNT proteins [37,38], suggesting that signals from neighboring epithelium are necessary for maintenance of DP function (see Figure 1B).

Not surprisingly, these extensive epithelial–mesenchymal interactions require the activities of secreted ligands that allow communication between different cell types. WNT signals play key roles at the initiation of embryonic hair follicle development [39–42]. Fibroblast growth factor proteins and the tumor necrosis factor family member ectodysplasin also promote epithelial placode formation, while Sonic hedgehog controls proliferation of both embryonic and postnatal hair follicle epithelium [30]. The BMP inhibitor Noggin is essential for hair follicle development [43], and acts in conjunction with WNT signaling and transforming growth factor β2 to control invasion of the dermis by hair follicle epithelial cells [44,45]. Differentiation of matrix cells toward either inner root sheath or hair shaft requires BMP signaling and numerous transcription factors [30,46–49]. The precise mechanisms by which these factors facilitate communication between cells of different types to produce an organ as complex as the hair follicle remain only partially understood.

## Hair Follicle Growth: The Role of SCs

The hair follicle undergoes cycles of growth and regression throughout life. At the onset of a new cycle of hair growth (anagen), signals from the DP are thought to stimulate the transient proliferation of epithelial SCs in the permanent bulge region of the follicle [50]. Bulge SC descendants give rise to all the epithelial layers of the regenerating hair follicle [51–55] (see Figure 1B). The extent to which these cells also contribute to epidermis in normal and pathological situations is currently not clear. Epithelial SCs coexist in the bulge with a population of melanocyte SCs that proliferates transiently at anagen onset and gives rise to differentiated melanocytes that populate the hair follicle bulb [56]. Hair graying in genetic mouse models and in humans is associated with loss of the melanocyte SCs [57]. Forced expression of a stabilized form of the WNT pathway effector â-catenin in hair follicle epithelial SCs causes the onset of a new cycle of growth of pigmented hair, suggesting that WNT signaling is critical for epithelial SC proliferation, and that secreted factors downstream of β-catenin can activate melanocyte SCs [58–60].

Several recent reports have identified cell populations in the skin that appear to be capable of differentiating into cell types other than hair follicle epithelium and epidermis, raising the exciting possibility that skin SCs could be used to regenerate organs beside the skin and hair follicles. For example, neural crest derivatives [14] and cells expressing a Nestin–green fluorescent protein (GFP) transgene [61] have been isolated from the bulge regions of vibrissa (whisker) follicles and can differentiate in culture into neurons, smooth muscle cells, and melanocytes. Multipotent cells termed skin-derived precursor cells have also been derived from the dermis of adult skin, and several lines of evidence suggest that the endogenous niche for these cells is the hair follicle DP [13]. In a separate study, clonal cell lines established from dissected rat vibrissa DPs and dermal sheaths had extended proliferative capacities and could differentiate into fat- and bone-related lineages [62]. Although it is possible that the skin-derived precursor cells and dissected vibrissa preparations could have been contaminated with non-DP cells, these data suggest that DP cells not only possess remarkable inductive capacities but also reside in a niche provided by the surrounding follicular cells that enables them to maintain a large proliferative capacity and the ability to differentiate into multiple cell types.

Relatively few cells reside in specialized niches such as the hair follicle DP and bulge, making molecular analyses of pure populations of these cells difficult. Recently, however, hair follicle bulge epithelial SCs have been isolated by utilizing a specific promoter to target GFP to adult bulge epithelium [52], or by taking advantage of the rarely cycling properties of epithelial bulge cells to target GFP expression to this population [55]. These approaches permit the use of fluorescence-activated cell sorting (FACS) to isolate and transcriptionally profile pure bulge epithelial SC populations.

In the current issue of *PLoS Biology*, Rendl et al. [63] take similar approaches to systematically analyze the transcriptional profile of the DP and four surrounding cell types: dermal fibroblasts, melanocytes, epithelial matrix cells, and outer root sheath cells, from mouse hair follicles four days after birth. At this time point, embryonic hair follicles have reached a late stage in their differentiation and are beginning to synthesize hair shafts. Rendl et al. marked each skin cell population with a unique combination of fluorescent tags, using a clever mix of cell-type-specific transgenic expression of red and green fluorescent proteins, together with immunolabeling of specific antigens. Each of the different cell populations was found to possess a distinct molecular signature. These experiments identified genes not previously associated with hair follicles, and revealed cell-type-specific expression for several genes affected in hair disorders.

Importantly, the results of this study support and extend previous observations that the DP expresses genes originally associated with neural SCs [13]. However, Rendl et al. find that the DP transcriptional profile is clearly distinct from that of neural SCs, neural crest, or melanocytes, and is most similar to that of dermal fibroblasts. These data support the concept that DP cells and dermal fibroblasts arise from similar precursors. Consistent with this view, use of a Hoxb6-Cre transgene to label derivatives of lateral plate mesoderm resulted in apparent marking of ventral trunk DPs as well as ventral trunk dermal fibroblasts, suggesting that at least some DP cells have common origins with neighboring dermal fibroblasts ([11]; S. I. Candille and G. S. Barsh, personal communication). Thus, differences in gene expression in DP cells compared with neighboring dermis may reflect the influences of follicular matrix and outer root sheath cells, rather than being the result of a distinct embryonic origin of the DP. Interestingly, WNT signaling and inhibition of BMP signaling are key elements in induction and maintenance of both hair follicles [39–43] and neural crest [1], and are featured in the DP signature. The re-utilization of these pathways in the complex signaling environment of the hair follicle may account in part for the unusual gene expression pattern in the DP, for the remarkable inductive properties of DP cells, and perhaps for their apparent ability to maintain multipotency.

The approach employed by Rendl et al. has produced a remarkably comprehensive picture of gene expression in different components of the skin and hair follicles in the early postnatal period. These data will likely form the basis for a multitude of further investigations into the molecular nature of mesenchymal–epithelial interactions in the hair follicle, and the functions of hair-follicle-expressed genes in both normal development and disease. Similar approaches could be employed to begin to address outstanding questions in skin and appendage biology. Profiling of different follicular compartments during the hair growth cycle may identify putative inductive signals produced by the DP, and could provide clues as to how cycling activity of epithelial and melanocyte SCs is coordinated. Profiling of different cell compartments in various developing appendages might begin to reveal the molecular mechanisms by which apparently similar sets of signaling pathways direct the formation of diverse organs. As the basic mechanisms of hair follicle development and hair growth are conserved between mice and humans [30], these studies are directly relevant to human hair follicle biology. However, some important aspects of human hair biology, such as the follicular response to androgens at puberty and in male pattern baldness, are absent in mice [64]. The development of antibody-based multicolor labeling systems to isolate pure populations of human hair follicle cells would therefore be a valuable additional tool for investigation of human hair disease.

## Abbreviations

BMP: bone morphogenetic protein
DP: dermal papilla
E[number]: embryonic day [number]
GFP: green fluorescent protein
SC: stem cell
